# Intravascular Large B-Cell Lymphoma Case Report: A Diagnostic Challenge

**DOI:** 10.7759/cureus.73704

**Published:** 2024-11-14

**Authors:** Ty M Moore, Aung M Tun, Sanjana Mullangi, Daniel Farrell, Joseph G Bennett

**Affiliations:** 1 Internal Medicine, University of Kansas Medical Center, Kansas City, USA; 2 Hematologic Malignancies and Cellular Therapeutics, University of Kansas Health System, Westwood, USA; 3 Hematologic Malignancies and Cellular Therapeutics, University of Kansas Cancer Center, Kansas City, USA; 4 Pathology and Laboratory Medicine, University of Kansas Medical Center, Kansas City, USA; 5 Hematologic Malignancies and Cellular Therapeutics, University of Kansas Health System, Kansas City, USA

**Keywords:** extranodal lymphomas, hematologic malignancies, intravascular large b-cell lymphoma, muscle biopsy, non-hodgkin lymphomas

## Abstract

A 66-year-old female presented to the hospital for evaluation of multiple strokes over a three-month period. The patient underwent extensive testing to evaluate for autoimmune vasculitis and other hypercoagulable entities that were negative. Bone marrow and lymph node biopsies showed no evidence of lymphoma. An excisional muscle biopsy was then performed, which demonstrated occasional small blood vessels containing atypical CD20+ lymphoid cells, and a diagnosis of intravascular large B-cell lymphoma (IVLBCL) was rendered. The patient underwent systemic chemotherapy along with CNS-directed therapy, followed by a consolidative autologous stem cell transplant. Here, we will discuss an unusual case of IVLBCL, some of the complications that can arise when diagnosis is delayed, and some of the key principles of diagnostic workup and management.

## Introduction

Intravascular large B-cell lymphoma (IVLBCL) is a rare subtype of extranodal LBCL characterized by the proliferation of lymphoma cells within the lumens of small-to-medium-sized blood vessels [1]. The malignancy often presents with nonspecific signs and symptoms, including fever and involvement of the central nervous system (CNS), with the most frequent manifestations being sensory and motor deficits, various skin manifestations, and the absence of lymphadenopathy [2]. Given that it primarily involves blood vessels and can display a metastatic clinical presentation, it is considered a disseminated disease at diagnosis. Due to its unusual presentation and clinically aggressive behavior, it often results in a delay in diagnosis with associated complications.

## Case presentation

**Figure 1:**
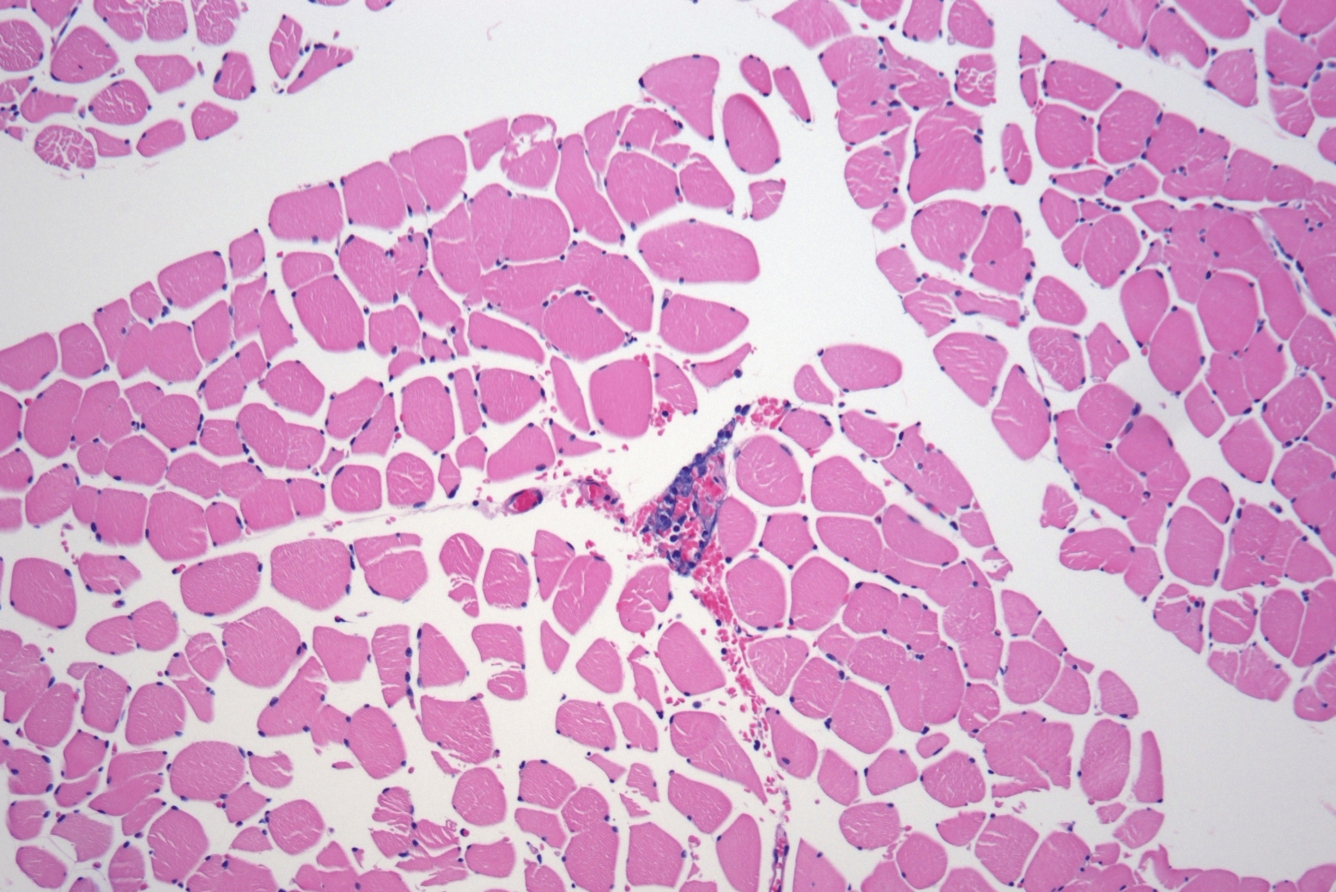
Muscle biopsy (100× magnification): H&E-stained section of skeletal muscle from the right leg showing the presence of atypical cells within a vessel The cells are notably confined to the lumen with no extension into surrounding tissue.

**Figure 2:**
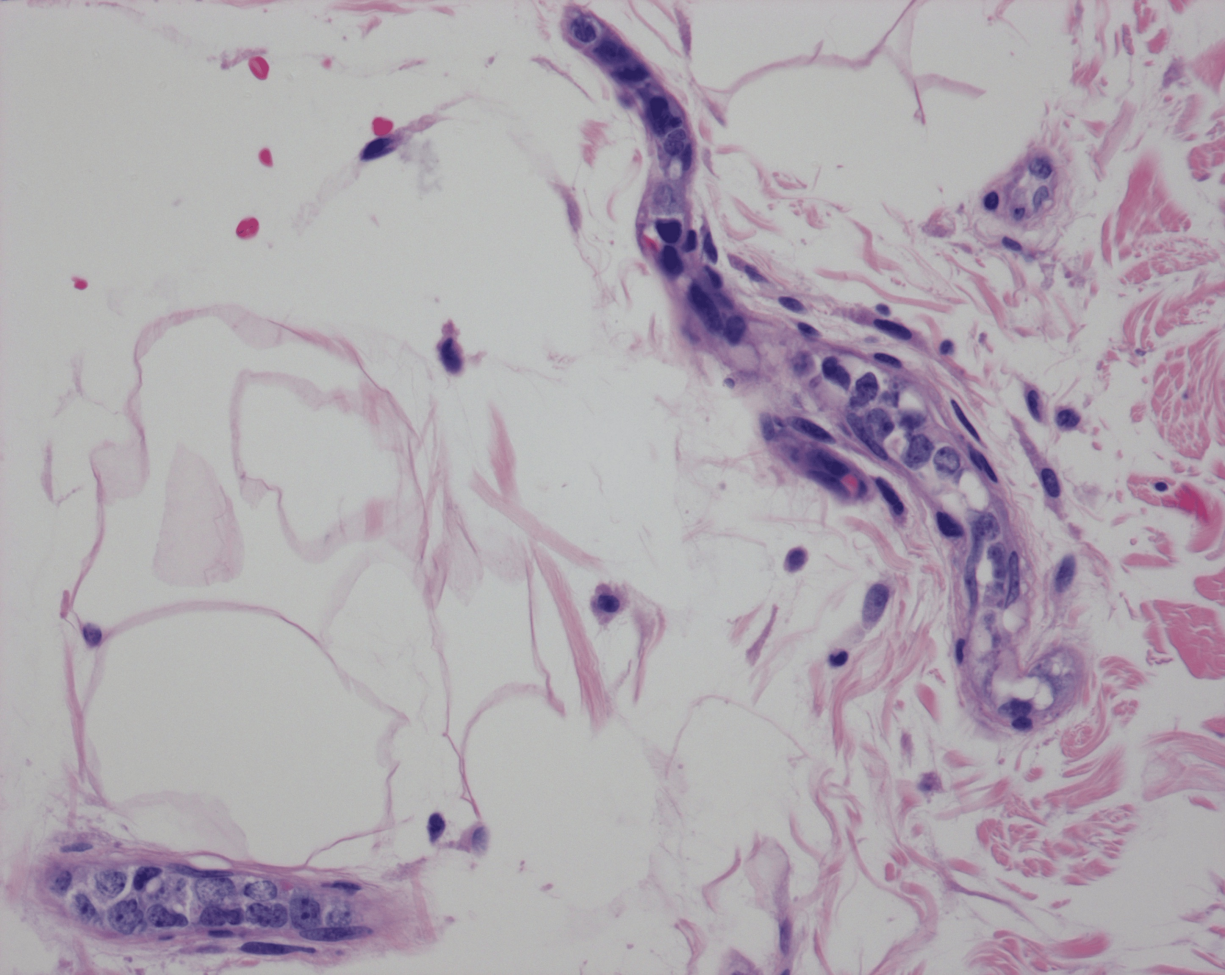
Skin biopsy (400× magnification): high power view of an H&E-stained section of skin from the right leg showing atypical cells confined to vessels The cells are intermediate-to-large in size with irregular nuclear contours, dispersed chromatin, multiple prominent nucleoli, and scant cytoplasm.

**Figure 3:**
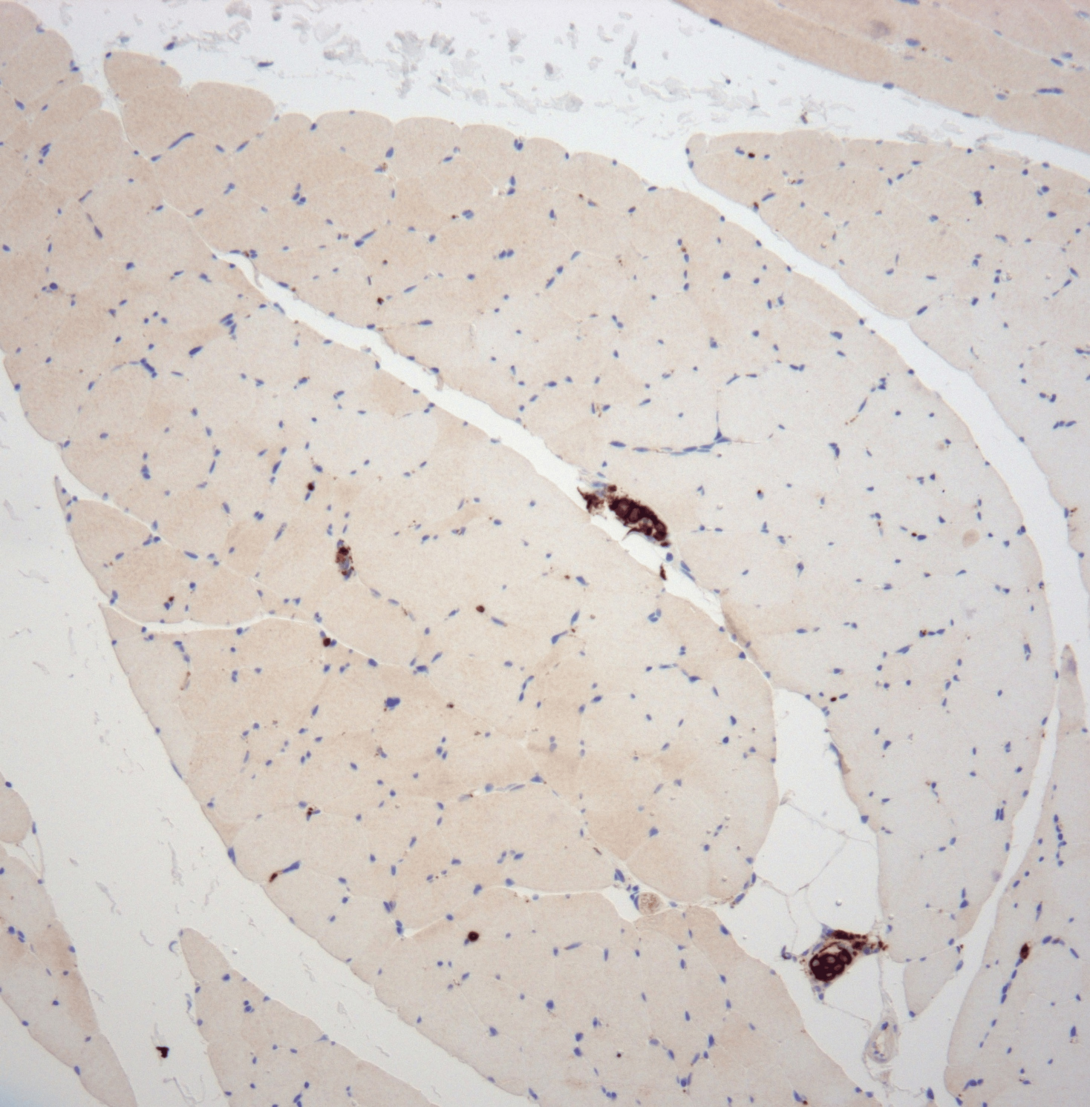
Muscle CD20 (100× magnification): immunohistochemical stain for CD20 performed on skeletal muscle tissue The atypical cells within vessels exhibit strong, uniform CD20 expression, consistent with B-cell phenotype.

**Figure 4:**
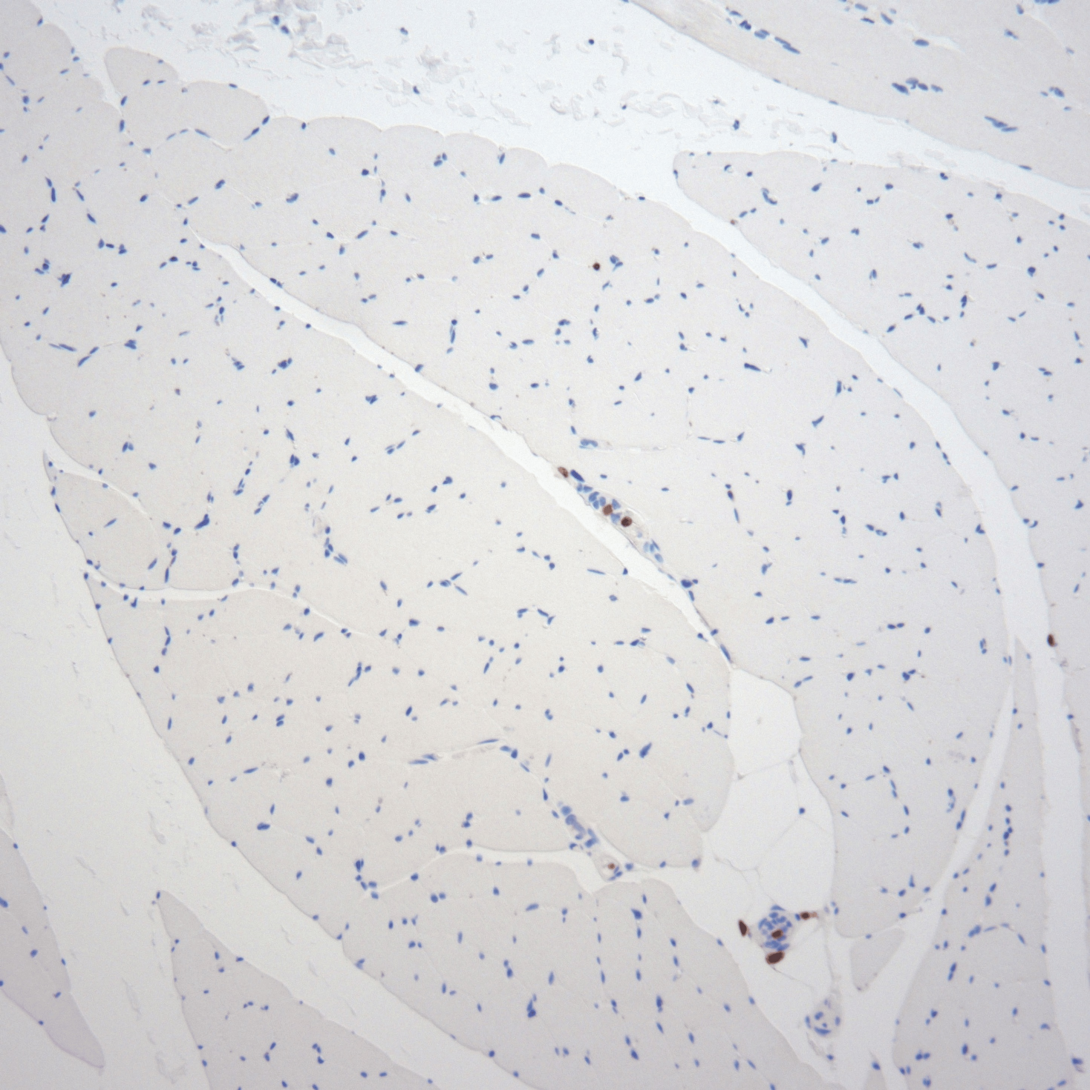
Muscle CD3 (100× magnification): immunohistochemical stain for CD3 performed on skeletal muscle tissue in the same areas seen in Figure 3 CD3, a T-cell marker, is negative in the large, atypical cells and positive in only a few small lymphocytes.

A 66-year-old female with hypertension, hyperlipidemia, hypothyroidism, and depression was admitted from a rehab facility with worsening functional decline with numerous falls, weakness, increasing confusion, and a history of two recent ischemic strokes. Her physical examination revealed worsening of her chronic left-sided hemiparesis. Magnetic resonance imaging (MRI) of the brain revealed extensive multifocal areas of abnormal hyperintense FLAIR and diffusion signal involving bilateral subcortical and periventricular cerebral white matter, posterior corpus callosum, and bilateral cerebellum. The findings demonstrated progression compared to prior MRI exams over the two previous months, where she had presented with left facial droop, aphasia, and weakness. She had undergone an ischemic stroke workup during her initial hospitalization, which included a transesophageal echocardiogram that was negative for patent foramen ovale or thrombus. Her previous MRI/MRA head imaging during her hospitalizations demonstrated numerous foci of abnormal signal hyperintensity on diffusion-weighted imaging. The findings were consistent with multiple acute to early subacute infarcts within small vessel territories. There was no evidence of acute large territorial infarct. With these prior findings along with her new MRI results, a wide differential was being considered, including vasculitis, autoimmune demyelinating process, and cerebral autosomal dominant arteriopathy with subcortical infarcts and leukoencephalopathy (CADASIL). Her autoimmune and infectious workups were unrevealing. Hypercoagulable workup was unrevealing. Neurology was consulted for possible CADASIL as an explanation for her multiple strokes in the absence of significant vascular disease. An excisional muscle biopsy (Figure 1) and a random deep skin biopsy (Figure 2) were performed from the right lower extremity to evaluate for CADASIL. The biopsies demonstrated occasional small blood vessels containing atypical CD20+ hematolymphoid cells (Figure 3) and no uptake of CD3 immunohistochemical stain in the large, atypical hematolymphoid cells (Figure 4), most consistent with the presence of IVLBCL. Her initial lab work demonstrated normocytic anemia of around 10 g/dL with a normal comprehensive metabolic panel. She had a mildly elevated erythrocyte sedimentation rate of 33 and a C-reactive protein of 28, along with an elevated lactate dehydrogenase of 330 at the time of diagnosis. A lumbar puncture demonstrated a rise in lymphocytes present in the cerebrospinal fluid; however, cytologic examination and flow cytometric analysis of the CSF were negative for the presence of malignant cells. Bone marrow biopsy did not reveal any definitive morphologic, flow cytometric, or immunohistochemical evidence of involvement by lymphoma. She had positron emission tomography/computed tomography (PET/CT) imaging performed that demonstrated inflammatory changes in the right quadriceps muscle where the patient had just had a muscle biopsy performed, but no other significant hypermetabolic activity or adenopathy was identified.

She was treated with rituximab, cyclophosphamide, doxorubicin hydrochloride, vincristine sulfate, and prednisone for two cycles (R-CHOP). Her clinical condition continued to decline with worsening performance status, and she was readmitted to the hospital for a neutropenic fever. Repeat PET/CT imaging during the admission demonstrated a new hypermetabolic left hilar mass. Bronchoscopy was performed, and a biopsy was obtained from the mass, revealing small yeast forms suggestive of histoplasmosis otherwise negative for atypical hematolymphoid cells or evidence of lymphoma. Histoplasmosis was treated with a three-month course of itraconazole. Due to a lack of clinical response with non-CNS directed chemoimmunotherapy, she was subsequently treated with two cycles of methotrexate plus cytarabine and one cycle of methotrexate plus rituximab as her intravascular lymphoma was suspected to be the culprit of the previous strokes. The interim MRI of the brain and PET/CT imaging following the CNS-directed therapy were negative. She had marked clinical improvement in her performance status and no recurrent ischemic strokes after the completion of methotrexate and cytarabine. As a consolidative strategy, the patient underwent autologous stem cell transplant using standard carmustine, etoposide, cytarabine, and melphalan (BEAM) conditioning regimen. The patient continues to routinely follow up with hematology since her treatment approximately 16 months ago. Her PET/CT scans and MRI imaging of the head have remained negative for disease recurrence. Flow cytometry of peripheral blood has remained negative for evidence of lymphoma as well. She continues to have a complete response to date without evidence of any new systemic or CNS abnormalities. She does continue to have chronic residual left-sided weakness from previous strokes, but her symptoms have improved with physical therapy and home health assisting her at home.

## Discussion

IVLBCL is a rare type of extranodal LBCL with an incidence of around one per 1,000,000 [3]. It was first described in the literature in 1959 when researchers believed that the malignant cells may be derived from endothelium as they were discovered to only be present in the intraluminal space of vasculature [3]. However, over time, we would learn that this behavior, along with the absence of malignant lymphoma cells within lymph nodes, is a hallmark of the disease [3].

Diagnosing IVLBCL can be significantly challenging for several reasons. IVLBCL has the ability to involve multiple organ systems and can often mimic a number of clinical presentations, such as hepatobiliary infections [4], hypersensitivity pneumonitis [5], and dermatological conditions [6]. Another diagnostic challenge is PET/CT scans, which can often be unrevealing in IVLBCL [7]. The definitive diagnosis often comes through deeper skin or soft tissue biopsies that are sometimes taken at random to obtain the involved blood vessels, primarily in the subcutaneous fat [8]. The diagnosis can then be made when a biopsy reveals the atypical lymphoma cells within the visualized vasculature. Although random skin biopsies are still somewhat controversial, experts recommend that skin biopsies should include at least three different sites to improve overall yield [9].

IVLBCL commonly presents with symptoms related to the involved organ, mainly neurological or cutaneous sites, in Western countries [10], whereas the disease presents as hemophagocytic syndrome in Asian countries [11]. The clinical features in the classical variant, occurring about 70-75% of the time, include a spectrum of heterogeneous symptoms, including fever of unknown origin, pain, constitutional symptoms, organ-specific local symptoms, and multi-organ failure. Fever can occur in at least half of patients as a systemic manifestation. Cutaneous involvement may be present in about 40% of patients at presentation with a wide variety of morphologies [10]. Neurologic symptoms can occur with variable symptoms, including motor and sensory deficits or neuropathies [12]. Neurolymphomatosis, especially in relapsing cases, can be seen with IVLBCL more frequently compared to other lymphomas where patients have sensorimotor weakness and neuropathic pain [13]. MRI is paramount in diagnostic workup in patients presenting with CNS symptoms [13]. IVLBCL frequently involves the CNS but is rarely limited to this system. Neurological imaging aids in diagnosis, especially when ischemic foci are anticipated. Because of this finding, vasculitis is often an important differential diagnosis that needs to be considered. 

The cutaneous variant of IVLBCL encompasses about 25% of the total cases with single or multiple skin lesions as the presenting complaint [10]. The cutaneous variant is more common in Western countries historically [10]. The cutaneous variant clinical presentation is less aggressive compared to the classical variant and is mostly seen in females and younger patient populations [2]. The overall survival for this variant is significantly longer than the classical variant [2].

The clinical presentation of the hemophagocytic syndrome variant typically presents like hemophagocytic lymphohistiocytosis with bone marrow involvement, associated fever, hepatosplenomegaly, and various cytopenias. This variant is more commonly seen in Asian countries and commonly referred to as the “Asian” variant. Unfortunately, the variant often displays a rapid and aggressive onset with a poor median survival time [14].

Given the rarity of IVLBCL, randomized controlled trials to guide systemic therapy do not yet exist. The literature currently available is used to guide clinical decision-making, which comes primarily from case reports and retrospective studies. Anthracycline-based chemotherapy has demonstrated response rates of >60% and is the most commonly utilized for systemic therapy [14]. The most often used treatment regimen is R-CHOP [10]. When patients are found to have CNS involvement, they require additional treatment, usually with intrathecal or systemic methotrexate and possibly radiation therapy, as traditional chemoimmunotherapy does not have appropriate CNS penetration [15]. With the current recommended treatment and guidelines, the estimated two-year overall survival is approximately 66% [16].

## Conclusions

Given its nonspecific clinical presentation and rareness, intravascular lymphoma is challenging to suspect and diagnose. The overlap of challenges in clinical, histological, and radiological aspects that IVLBCL has with different disease entities requires a multidisciplinary approach to the workup to reach a definitive diagnosis. Due to IVLBCL being clinically aggressive and causing numerous complications when the diagnosis is delayed, it should be considered in the differential when a patient presents with constitutional symptoms with neurologic findings and/or skin lesions. Additional research is needed to better understand disease biology and tailor treatment approaches to improve outcomes for patients with IVLBCL.
